# Multimodality Evaluation of Aortic Insufficiency and Aortitis in Rheumatologic Diseases

**DOI:** 10.3389/fcvm.2022.874242

**Published:** 2022-04-12

**Authors:** Eunjung Choi, Lena M. Mathews, Julie Paik, Mary C. Corretti, Katherine C. Wu, Erin D. Michos, Allison G. Hays, Monica Mukherjee

**Affiliations:** ^1^Dartmouth-Hitchcock Medical Center, Heart and Vascular Center, Lebanon, NH, United States; ^2^Division of Cardiology, Johns Hopkins University, Baltimore, MD, United States; ^3^Division of Rheumatology, Johns Hopkins University, Baltimore, MD, United States

**Keywords:** aortic insufficiency (AI), autoimmune disease (AD), rheumatologic diseases, aortitis, ankylosing spondylitis, Libman-Sacks endocarditis, Takayasu arteritis

## Abstract

Aortic insufficiency is commonly observed in rheumatologic diseases such as ankylosing spondylitis, systemic lupus erythematosus, antiphospholipid syndrome, Behçet's disease, granulomatosis with polyangiitis, and Takayasu arteritis. Aortic insufficiency with an underlying rheumatologic disease may be caused by a primary valve pathology (leaflet destruction, prolapse or restriction), annular dilatation due to associated aortitis or a combination of both. Early recognition of characteristic valve and aorta morphology on cardiac imaging has both diagnostic and prognostic importance. Currently, echocardiography remains the primary diagnostic tool for aortic insufficiency. Complementary use of computed tomography, cardiac magnetic resonance imaging and positron emission tomography in these systemic conditions may augment the assessment of underlying mechanism, disease severity and identification of relevant non-valvular/extracardiac pathology. We aim to review common rheumatologic diseases associated with aortic insufficiency and describe their imaging findings that have been reported in the literature.

## Introduction

Aortic insufficiency (AI) is an important cardiac manifestation observed in many rheumatologic and systemic diseases. In the Euro Heart Survey, about 4% of AI cases were thought to be due to an underlying inflammatory condition (1). The aortic valve can be insufficient due to a primary valve pathology (leaflet destruction, prolapse or restriction) or secondary to annular dilatation or both. Both primary and secondary forms of AI have been described in rheumatologic diseases. Two-dimensional echocardiography remains the mainstay diagnostic tool of choice in the assessment of AI; however, other imaging modalities such as computed tomography (CT), magnetic resonance imaging (MRI) and Fluorine-18 fluorodeoxyglucose positron emission tomography (FDG-PET) are also considered useful tests, especially when there is a concern for concomitant aortitis. Observational studies have reported high accuracy of cardiac CT and MRI derived classification of AI mechanism when compared to surgical inspection (2, 3). High spatial resolution data of the aortic valve and root provided by cardiac CT and multiplanar reconstructed images have been used to determine feasibility of aortic valve repair (4). Cardiac MRI is often used as the gold standard for measurements of left ventricular function, mass and volume. It is also frequently utilized for accurate quantification of AI by using phase-contrast velocity mapping (5, 6). Quantification of AI using cardiac MRI has been shown to have significant association with clinical outcomes such as symptom development and need for surgery (7). CT and MRI images of the aortic valve during diastole allow planimetric measurement of the regurgitant orifice in moderate or severe AI, although visualization on CT can be limited by concomitant aortic stenosis or calcification (3, 8). The use of advanced imaging in the assessment of AI is well-reviewed in the current literature, (9–12) but there is little information about specific findings related to underlying rheumatologic diseases. Ankylosing spondylitis, systemic lupus erythematosus, and antiphospholipid syndrome often involve primary valvular pathology leading to AI. In vasculitides such as Behçet's Disease, granulomatosis with polyangiitis, Takayasu arteritis, giant cell arteritis, Cogan's syndrome, and IgG-related aortitis, AI is mostly secondary to aortic inflammation and dilatation. In this review, we aim to discuss the rheumatologic diseases associated with AI and describe their characteristic imaging findings.

## Ankylosing Spondylitis

Ankylosing spondylitis is characterized by its strong association with *HLA B-27* (present in about 90% of patients), sacroilitis, enthesitis, uveitis, inflammatory bowel disease and psoriasis (13). Ankylosing spondylitis is more prevalent in men with a gender ratio of roughly 3.4:1 (14). The reported prevalence of AI in ankylosing spondylitis population ranges from 3.3 to 18% (15). A recent Dutch registry of patients with ankylosing spondylitis aged 50–75 years showed that they had an up to five times higher odds of having AI compared to controls after adjusting for age, sex and cardiovascular risk factors (16). A cross-sectional transthoracic echocardiography (TTE) study of 187 patients with ankylosing spondylitis showed that presence of AI was associated with increasing age, disease duration and a history of anterior uveitis (17). A recent observational study showed that *HLA B-27* positivity was associated with increased aortic root diameter index after adjusting for age, sex and cardiovascular risk factors (18). However, there was no association between *HLA B-27* positivity and AI. In a recent study, ankylosing spondylitis patients had a higher risk of developing valvular heart disease and undergoing valve replacement surgery compared to those without the disease (19). Cases of thoracic and abdominal aortic aneurysm in ankylosing spondylitis have been reported but the incidence of aortic dissection is unclear (20–22).

Histopathological findings of the affected aortic valve, root and sinuses include fibrous tissue deposition in the adventitia and intima resulting from platelet aggregation and fibroblast activation (23). It is thought that inflammation and subsequent dilatation of the aortic root in ankylosing spondylitis eventually lead to fibrotic thickening and shortening (i.e., inward rolling) of the aortic cusps and increased aortic stiffness (24, 25). Characteristic echocardiographic findings such as aortic root thickening (defined as wall thickness > 2.2 mm), nodular aortic valve thickening (defined leaflet thickness > 2 mm in two or more cusps or in one cusp with valve insufficiency) and subaortic bump (defined as an aorto-mitral junction length and height of >7.7 and 3.2 mm, respectively) were demonstrated in a case series of 44 patients (26). Subaortic bump caused by fibrotic changes can also affect the anterior mitral leaflet resulting in mitral regurgitation, but mitral valve involvement is rare in ankylosing spondylitis (27). A small explorative study looking at myocardial tissue characterization using cardiac MRI found that a subgroup of ankylosing spondylitis patients had a typical pattern of late gadolinium enhancement in the mid wall to subepicardial layer, similar to previously reported findings in other autoimmune diseases (28). The same study also found that myocardial extracellular volume, quantified by T1 mapping, was associated with disease activity.

## Systemic Lupus Erythematosus and Antiphospholipid Syndrome

Systemic lupus erythematosus (SLE) is a chronic autoimmune disorder of unknown cause. AI is a common valvular abnormality seen in patients with systemic lupus erythematosus (SLE), after mitral and tricuspid insufficiency (29). One of the cardiac manifestations associated with SLE and antiphospholipid syndrome (APLS) is Libman-Sacks endocarditis, also known as non-bacterial thrombotic endocarditis, which is often found on the left sided valves and related to valve insufficiency (30). Vegetations associated with Libman-Sacks endocarditis are frequently located at the tip or mid-portion of the leaflets but can involve the annulus or subvalvular apparatus (31, 32). Libman-Sacks vegetations are found in roughly 1 in 10 patients with SLE and in those with longer disease duration, higher severity, and positive antiphospholipid antibodies (present in 30–40% of SLE patients) (33). Due to its association with APLS, patients with both SLE and Libman-Sacks endocarditis are also at higher risk of thromboembolism. In a study of 69 SLE subjects, patients with Libman-Sacks endocarditis had 11% incidence of cerebrovascular accident and 12% mortality during a 5-year follow-up (34).

The vegetations are thought to be due to the formation of fibrin-platelet thrombi as well as deposition of immunoglobulins and complements, both of which ultimately result in valve fibrosis, distortion and dysfunction (35, 36). There are more valvular lesions found in patients with APLS secondary to SLE compared to those with primary APLS suggesting additional SLE-related immunologic factors may play a pathogenic role (37).

**Figure 1:**
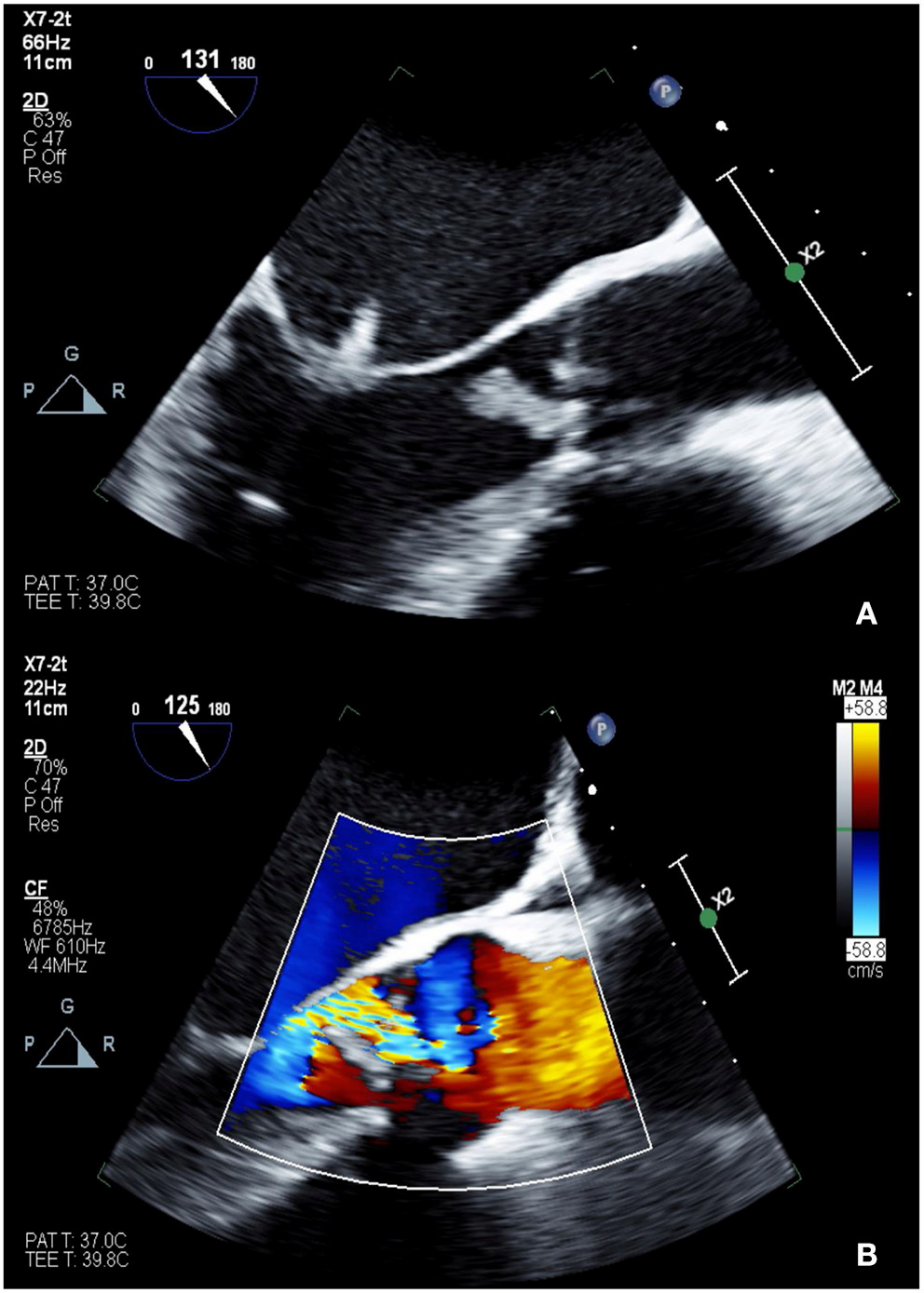
TEE showing 2D (A: top) and color Doppler (B: bottom) images of Libman-Sacks endocarditis involving the left-sided valves **(A)** and severe eccentric aortic insufficiency **(B)**. The patient was found to have positive lupus anticoagulant as well as high titers of anti-cardiolipin IgM and anti-beta-2 glycoprotein I IgM, consistent with triple positive APLS.

Roldan et al. showed that transesophageal echocardiography (TEE) is superior to TTE in detecting Libman-Sacks endocarditis and that 3D TEE is better than 2D in characterizing lesions (31, 32). By echocardiographic evaluation, the vegetations have been described as various sizes of irregularly shaped, sessile, and homogeneously echodense nodularities (Figure 1) (32). These lesions typically affect both sides of the valve surface. Based on the appropriate use criteria, cardiac CT may be an alternative non-invasive diagnostic test to visualize valvular vegetations in suspected Libman-Sacks when TTE is inconclusive and TEE is contraindicated (38). Differentiation between Libman-Sacks endocarditis and infective endocarditis by echocardiographic imaging alone, is difficult as there are no pathognomonic features distinguishing the two entities. Clinical diagnosis is generally made when there is an absence of systemic infection in a patient at risk for Libman-Sacks endocarditis. A recent case report suggested that cardiac MRI could be used to differentiate Libman-Sacks endocarditis from valve thrombosis and infective endocarditis by confirming increased T2-weighted signal and increased signal intensity on delayed hyperenhancement (39). However, sensitivity and specificity of these findings have not been studied broadly. In general, the relatively lower spatial resolution of cardiac MRI limits its sensitivity of tissue characterization of vegetations. FDG-PET may demonstrate increased uptake at the site of Libman-Sacks endocarditis but cannot distinguish this disease process from infective endocarditis (40). Libman-Sacks vegetations on imaging studies are indistinguishable from vegetations or valve thrombus. Therefore, patient's history, physical exam and extracardiac symptoms should be taken into consideration. Complete blood count, blood cultures and serologic tests should be part of evaluation in order to rule out other causes of vegetations such as infective endocarditis. In SLE and APLS, AI is primarily caused by the underlying valve pathology related to Libman-Sacks endocarditis. Although rare, there are reported cases of lupus aortitis leading to secondary AI, diagnosed by CT, MRI and PET findings along with high serum C-reactive protein levels (41). Lupus aortitis has also been associated with dissection, aneurysm and thrombus (42).

## Behçet's Disease

Behçet's disease is a chronic, relapsing vasculitis associated with *HLA-B51* and mutation of factor V Leiden (43). It most affects young individuals of Mediterranean and East Asian origin. It is diagnosed by multiple organ involvement including recurrent mucosal ulcers (oral and genital), skin manifestations, eye lesions (uveitis) and a positive pathergy test. Although uncommon, more severe complications of Behçet's disease include gastrointestinal, cardiac and vascular involvement of both venous and arterial vessels of all sizes. Cardiac involvement, such as valvular insufficiency, pericarditis, intracardiac thrombosis, myocarditis and myocardial infarction, was found to be the first manifestation of Behçet's disease in a third of the study cohort investigated by Geri et al. (44) Previously reported cases of AI in Behçet's disease patients were more frequently found in men than women and the average age of patients was 44 years (45). The prevalence of AI in Behçet's disease patients is reported to be around 16% (44). Histopathological examination of the aortic valve shows acute and chronic lymphoplasmocytic infiltration and fibrotic thickening (46). Characteristic echocardiographic findings of aortic valve disease related to Behçet's disease include aortic root dilatation, redundant coronary cusp motion or prolapse, sinus of Valsalva aneurysm with/without rupture, vegetation like mobile lesions, and periaortic echolucent spaces (pseudoaneurysm) mimicking abscess formation (47). The aortic valve pathology in Behçet's disease is complex and can mimic findings found in other diseases such as infective endocarditis or Libman-Sacks endocarditis. Culture data and serologic testing for other autoimmune processes should be obtained to rule out endocarditis. Echocardiography is a critical imaging modality in detecting post-operative complications which are unfortunately common in Behçet's disease patients undergoing valve replacement. Peri-operative immunosuppression is critical; however, prosthesis dehiscence, paravalvular leak, and periaortic pseudoaneurysm formation have been reported despite use of immunosuppressive treatment (48). Patients undergoing aortic valve replacement alone often need a second operation. Pre-operative features such as pseudoaneurysm at the ascending aorta and dissection into the interventricular septum have been found to be associated with higher rates of recurrent AI after first operation (49). Concurrent aortic root replacement with a homograft at the time of valve replacement is proposed as a preferred surgical method to prevent some of these post-operative complications (46).

Cardiac CT may be used for identification of periaortic pseudoaneurysm and other cardiac abnormalities such as endomyocardial fibrosis (low attenuation along the myocardium) and intracardiac thrombi (contrast filling defect) (50). CT coronary angiography can be used to look for coronary artery aneurysm with or without thrombus formation. Aortitis has also been reported in association with valvulitis in Behçet's disease. Invasive angiography was historically considered gold standard for diagnosis of aortitis, but advances in non-invasive imaging modalities such as CT and MR angiography have replaced invasive aortography with more rapid and accurate assessment of vascular abnormalities such as aneurysm, stenosis and calcification seen in “burnt out” aortitis (51). Invasive angiography is limited to detecting changes in luminal diameter, which occur in late stages of disease. Routine use of diagnostic invasive angiography is discouraged in Behçet's disease patients as there have been frequent reports of arterial puncture site complications including pseudoaneurysm formation and delayed rupture (52). Use of FDG-PET has shown FDG uptake in the aortic root, thoracic and/or abdominal aorta in Behçet's disease patients, consistent with active inflammation (48).

## Granulomatosis with Polyangiitis

Granulomatosis with polyangiitis (GPA) is a small to medium vessel necrotizing granulomatous vasculitis that affects the respiratory tract and the kidneys. It is associated with proteinase 3 anti-neutrophilic cytoplasmic antibody (PR3-ANCA) positivity. GPA affects the heart in 8–16% of cases at the time of diagnosis and in 4–25% of cases throughout the disease course (53). Cardiac complications in GPA include pericarditis, myocarditis, coronary arteritis and valvulitis. AI is the most frequently seen valve disease in this population (54). A retrospective study of 6,740 GPA patients found that 2% of the patients had AI (55). Histopathological findings from the affected aortic valve usually show polymorphonuclear micro-abscesses, foci of necrosis, and areas of fibrosis (54, 55).

By 2D echocardiography, aortic valve leaflets can appear thickened with focal restriction of cusps causing malcoaptation of the leaflets (56). Other valvular manifestations include perforation and vegetations which can resemble endocarditis (57). Although rare in GPA, aortic root and ascending aortic inflammation has been reported in a patient with known GPA presenting with severe AI and complete heart block (58).

## Takayasu Arteritis

Takayasu arteritis (TA) is an idiopathic large-vessel vasculitis involving the aorta and its branches as well as the pulmonary artery, which may result in stenosis, occlusion or aneurysm of the affected vessel (59). TA typically affects young women under 40 years of age (51). In the National Institutes of Health study of 60 patients with TA, 23% had aortic aneurysms, most commonly in the aortic arch/root, abdomen, then other thoracic segments (60). AI can result from aortic root dilatation in TA and was found in 13–44% of affected patients (61, 62).

Echocardiography may show normal aortic cusps and AI associated with root dilatation; however, there has been a case of aneurysm of the right sinus of Valsalva which developed 14 years after valve replacement in a Takayasu patient (63). Thickened aortic root and ascending aorta can be demonstrated on echocardiography, with complementary findings of diffuse wall thickening and gadolinium enhancement seen on CT and MRI, respectively (64). Cross sectional imaging studies should be used to evaluate the distal aorta and branch vessels that are not easily visualized on echocardiography. In a study of 85 TA patients who underwent CT angiography, 95% of patients were found to have aortic involvement and 5% of those patients had branch involvement (65). CT angiography may show wall thickening with mural enhancement and low-attenuation ring in early stages of active TA (65, 66). Late complications of TA such as large vessel aneurysm, stenosis, and occlusion may also be seen on CT angiography. Due to its strength in arterial wall characterization related to disease activity, CT angiography has an important role in monitoring disease severity and response to immunosuppressive therapy (67). Compared to CT, MRI has an advantage of obtaining multiplanar images without ionizing radiation. Disease activity in early vasculitis may be represented by increased wall thickness and mural contrast enhancement in T1-sequences as well as arterial wall edema in T2-sequences (68). FDG-PET plays an important role in the diagnosis and monitoring of TA by detecting active inflammation. It can diagnose early disease by showing inflammatory cell infiltration of the vessel wall, which occurs sooner than development of wall edema seen on MRI (69, 70). When compared with invasive angiography, FDG-PET correctly diagnosed 11 of 12 patients with active TA and all 6 patients with inactive TA, resulting 92% sensitivity and 100% specificity (71). PET findings can normalize after appropriate immunosuppressive therapy. On PET, vasculitic lesions have more intense FDG uptake (grade 2+ or 3+) and diffuse linear involvement as opposed to focal hot spots seen in atherosclerotic lesions (69). In patients with suspected aortitis, hybrid imaging with PET and either CT or MR angiography gives more precise anatomic localization of active disease.

## Other Vasculitides

Along with Takayasu arteritis, giant cell arteritis (GCA) is one of the leading causes of aortitis and aortic aneurysm. GCA is a large vessel vasculitis that generally affects individuals 50 years or older and aortitis is present at in at least 27% of cases (72). Aortic manifestations of GCA may include annuloaortic ectasia, thoracic and abdominal aortic aneurysm, dissection and/or AI (42). Patients with GCA are approximately 17 times more likely to develop thoracic aortic aneurysm compared to the general population (73). A population based cohort study of 204 patients with GCA found that the incidence of aortic aneurysm/dissection is high within the first year of diagnosis and increased after 5 years (74). In the same study, aortic aneurysm/dissection was associated with increased mortality with a hazard ratio of 3.4 (95% CI 2.2–5.4). These findings emphasize the importance of long-term surveillance and monitoring of aortic aneurysm formation in GCA patients. Cogan's syndrome is characterized by episodes of interstitial keratitis and vestibuloauditory dysfunction and aortitis occurs in up to 12% of patients (75). Cogan syndrome has a mortality rate of ~10%, largely due to cardiovascular complications such as ruptured aortic aneurysm, systemic vasculitis, myocardial infarction and heart failure (76). IgG4-related disease is a chronic autoimmune mediated fibroinflammatory condition that can affect many organ systems including the cardiovascular system. IgG4-aortitis is thought to be present in about 8% of IgG4-related disease patients and in 7–9% of non-infectious thoracic aortitis (77). In IgG4-aoritits, thoracic aortic involvement is twice as likely as abdominal aortic involvement (78). Aortic dilatation in these diseases can lead to secondary AI but the exact prevalence is unknown. Similar to the diagnostic approach used in Takayasu arteritis, aortic wall thickening, inflammation and vessel stenoses may be seen on CT, MRI, and PET imaging in these vasculitides (Table 1).

**Table 1:** Summary of imaging findings from rheumatologic diseases causing aortic insufficiency.

	**Echocardiography**	**CT**	**MRI**
Ankylosing spondylitis	• Valve thickening and calcification • Inward rolling of the aortic cusps • Subaortic bump • Can involve thickening of the anterior mitral leaflet and mitral regurgitation	• Annular dilatation and coaptation gap	• Aortic wall thickening, edema and/or mural contrast enhancement consistent with aortitis • Mid to subepicardial hyperenhacement
Libman-sacks endocarditis	• Various sizes of irregularly shaped, sessile, and homogenous echoreflectant lesions	• Various sizes of irregularly shaped valvular masses (characterization may be difficult)	• Increased T2-weighted signal and delayed hyperenhancement of the valve • Endocardial fibrosis consistent with myocarditis
Behçet's disease	• Thinning/elongation of leaflets • Redundant coronary cusp motion or prolapse • Vegetation like mobile lesions • Periaortic echolucent spaces (pseudoaneurysm) • Aortic root dilatation • Sinus of Valsalva aneurysm (rare)	• Low attenuation along the myocardium consistent with endomyocardial fibrosis • Periaortic pseudoaneurysm • Aortic wall thickening with mural enhancement • Intracardiac thrombi • Coronary artery aneurysm	• Aortic wall thickening, edema and/or mural contrast enhancement consistent with aortitis • Myocardial edema
Granulomatosis with polyangiitis	• Thickened leaflets and coal restriction of cusps causing malcoaptation • May involve valve perforation and vegetations	• Thickening of the aortic valve cusps	• Asymmetrical thickening of the aortic root and ascending aorta • Increased T2-weighted signal and delayed hyperenhancement of the valve and ascending aorta
Takayasu arteritis and other vasculitides	• Normal aortic cusps • Aortic annular dilatation • Thickening of the annulus and ascending aorta • Sinus of Valsalva aneurysm (rare)	• Aortic wall thickening with mural enhancement +/- involvement of aortic branches • Aneurysm, stenosis or occlusion of the aorta or its branches	• Aortic wall thickening, edema and/or mural contrast enhancement consistent with aortitis

## Conclusion

Many rheumatologic diseases result in aortic valve pathology, aortitis and subsequent AI. A comprehensive rheumatologic investigation should take place when there is high clinical suspicion for an underlying autoimmune disease and otherwise unexplained acute or chronic AI. A detailed history taking, physical examination as well as serologic testing should be part of the work up. TTE is a useful first-step diagnostic tool for assessment of AI and proximal aortic pathology, although it may not differentiate the underlying rheumatologic condition. When there is an eccentric jet of AI, additional imaging such as TEE and cardiac MRI can be used for more accurate assessment of disease severity. Cardiac CT can demonstrate other intra/extracardiac abnormalities such as intracardiac thrombus and periaortic pseudoaneurysm. FDG-PET is a useful diagnostic tool for detecting active inflammation in these rheumatologic diseases. It is important to consider involvement of the thoracic aorta and other large vessels and use supplemental imaging modality such as CT, MRI and PET to make the correct diagnosis. Additional research investigating the clinical features that predict the presence, severity and progression of AI in rheumatic diseases is much needed as early recognition of the valve dysfunction and associated aortopathy has crucial therapeutic and prognostic implications.

## Author Contributions

All authors listed have made a substantial, direct, and intellectual contribution to the work and approved it for publication.

## Conflict of Interest

The authors declare that the research was conducted in the absence of any commercial or financial relationships that could be construed as a potential conflict of interest.

## Publisher's Note

All claims expressed in this article are solely those of the authors and do not necessarily represent those of their affiliated organizations, or those of the publisher, the editors and the reviewers. Any product that may be evaluated in this article, or claim that may be made by its manufacturer, is not guaranteed or endorsed by the publisher.
